# Successful surgical treatment of postmyomectomy uterine diverticulum: a case report

**DOI:** 10.1186/s12905-023-02539-1

**Published:** 2023-08-03

**Authors:** Rina Kawatake, Aki Maebayashi, Haruna Nishimaki, Masaji Nagaishi, Kei Kawana

**Affiliations:** 1 Department of Obstetrics and Gynecology, Nihon University Hospital, 1-6 Kanda Surugadai Chiyoda-ku, Tokyo, 101-8309 Japan; 2 Department of Obstetrics and Gynecology, Nihon University School of Medicine, 30-1 Oyaguchi Kami-cho, Itabashi-ku, Tokyo, 173-8610 Japan; 3 Department of Pathology and Microbiology, Nihon University Hospital, 1-6 Kanda Surugadai Chiyoda-ku, Tokyo, 101-8309 Japan

**Keywords:** Hysteroscopy, Laparoscopy, Postmyomectomy, Surgical treatment, Uterine diverticulum

## Abstract

**Background:**

Uterine diverticulum is classified into congenital and acquired types. The acquired type is caused by caesarean scar syndrome, which occurs after caesarean section. There are no detailed reports on diverticulum after enucleation of uterine fibroids. Most cases are treated with hysteroscopy or laparoscopy, but a management consensus is lacking. We treated a patient with a uterine diverticulum that had formed after uterine fibroid enucleation by combining hysteroscopic and laparoscopic treatments.

**Case presentation:**

The patient was a 37-year-old Japanese woman, G1P0. A previous doctor had performed abdominal uterine myomectomy for a pedunculated subserosal uterine fibroid on the right side of the posterior wall of the uterus near the internal cervical os. Menstruation resumed postoperatively, but a small amount of dark-red bleeding persisted. MRI two months after the myomectomy revealed a diverticulum-like structure 3 cm in diameter, communicating with the uterine lumen, on the right side of the posterior wall of the uterus. Under suspicion of uterine diverticulum after uterine fibroid enucleation, the patient sought treatment at our hospital approximately four months after the myomectomy. Through a flexible hysteroscope, a 5-mm-diameter fistula was observed in the posterior wall of the uterus, and a contrast-enhanced pocket, measuring approximately 3 cm, was located behind it. Uterine diverticulum following enucleation of a uterine fibroid was diagnosed, and surgery was thus deemed necessary. The portion entering the fistula on the internal cervical os side was resected employing a hysteroscope. Intra-abdominal findings included a 4-cm mass lesion on the posterior wall on the right side of the uterus. The mass was opened, and the cyst capsule was removed. A 5-mm fistula was detected and closed with sutures. Resuturing was not performed after dissection of the right round ligament due to tension. The postoperative course has been good to date, with no recurrence.

**Conclusion:**

Uterine diverticula after myomectomy may be treated with a combined laparoscopic and hysteroscopic approach, similar to caesarean scar syndrome.

## Background

Uterine diverticulum is classified into congenital and acquired types. Congenital uterine diverticulum, caused by Müllerian duct anomalies, is rare, while acquired uterine diverticulum due to surgical trauma mostly results from caesarean section [1]. Symptoms include abnormal bleeding, dysmenorrhea, and pelvic pain, and this condition can even lead to infertility [2][3]. Treatment selection is based on factors such as infertility and the desire to preserve the uterus, and the method of treatment is based on the size of the diverticulum, the thickness of the residual muscular layer, and other anatomical factors [2]. While there are several reports on surgical treatment methods such as laparoscopy, hysteroscopy, combinations of the two, and vaginal procedures [4], a gold standard has yet to be established. The number of myomectomy procedures has increased in recent years, although reports of uterine diverticulum after myomectomy are extremely rare. To our knowledge, this is the first case not associated with pregnancy to be reported.

## Case presentation

**Fig. 1:**
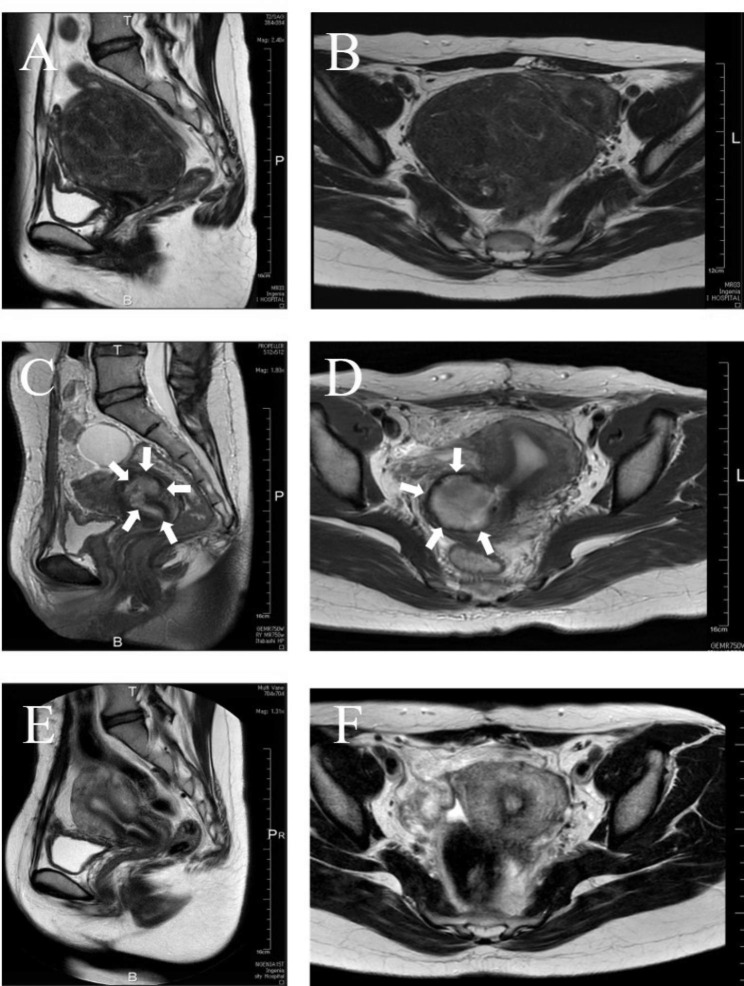
MRI findings. Before myomectomy, a 13-cm subserosal fibroid is present on the posterior wall of the lower part of the uterine body. **A**: Sagittal T2-weighted MRI; **B**: Axial T2-weighted MRI. After myomectomy, a 3-cm haematoma is observed at the enucleation site (white arrow). **C**: Sagittal T2-weighted MRI; **D**: Axial T2-weighted MRI. After haematoma removal and uterine wall formation, strong anteflexion of the uterus diminished without haematoma recurrence. **E**: Sagittal T2-weighted MRI; **F**: Axial T2-weighted MRI

The patient was a 37-year-old Japanese woman, G1P0 (one ectopic pregnancy; due to left fallopian tube pregnancy, left salpingectomy had been performed by laparotomy.). The patient had consulted a previous doctor with complaints of lower abdominal pain. A subserosal uterine fibroid 9 cm in diameter was found in the posterior wall of the uterus [Fig. 1A and B] and was removed after administration of a gonadotropin-releasing hormone antagonist. The patient’s Hb was 8.4 g/dL, and she had anaemia due to menorrhagia at the initial presentation. Thus, she had been taking a gonadotropin-releasing hormone antagonist (Religolix; Relumina tablets 40 mg) for 95 days before surgery. Just prior to the operation, the fibroid was approximately 9 cm in size, and no clear shrinkage was obtained. The last dose of GnRHa at the previous hospital had been taken 12 days before myomectomy.

The surgical findings included a 9-cm pedunculated subserosal uterine fibroid that had developed from the right side of the posterior wall of the uterus near the internal cervical ostium. This fibroid was also found to have grown into the retroperitoneal space. The fibroid stalk was clamped and cut, and the stump was ligated. The injured muscular layer was sutured in two layers with synthetic absorbable thread (Vicryl Ethicon), and abdominal uterine myomectomy was performed. The histopathological diagnosis was cellular leiomyoma. Menstruation resumed on the 25th day after the procedure, but small amounts of dark-red bleeding (spotting) persisted. Magnetic resonance imaging (MRI) approximately two months after the myomectomy revealed a 3-cm diverticulum-like structure communicating with a 5-mm fistula and the uterine lumen on the right side of the posterior wall of the uterus. Under suspicion of uterine diverticulum developing after enucleation of a uterine fibroid, the patient was referred to our hospital nearly four months after the myomectomy.

**Fig. 2:**
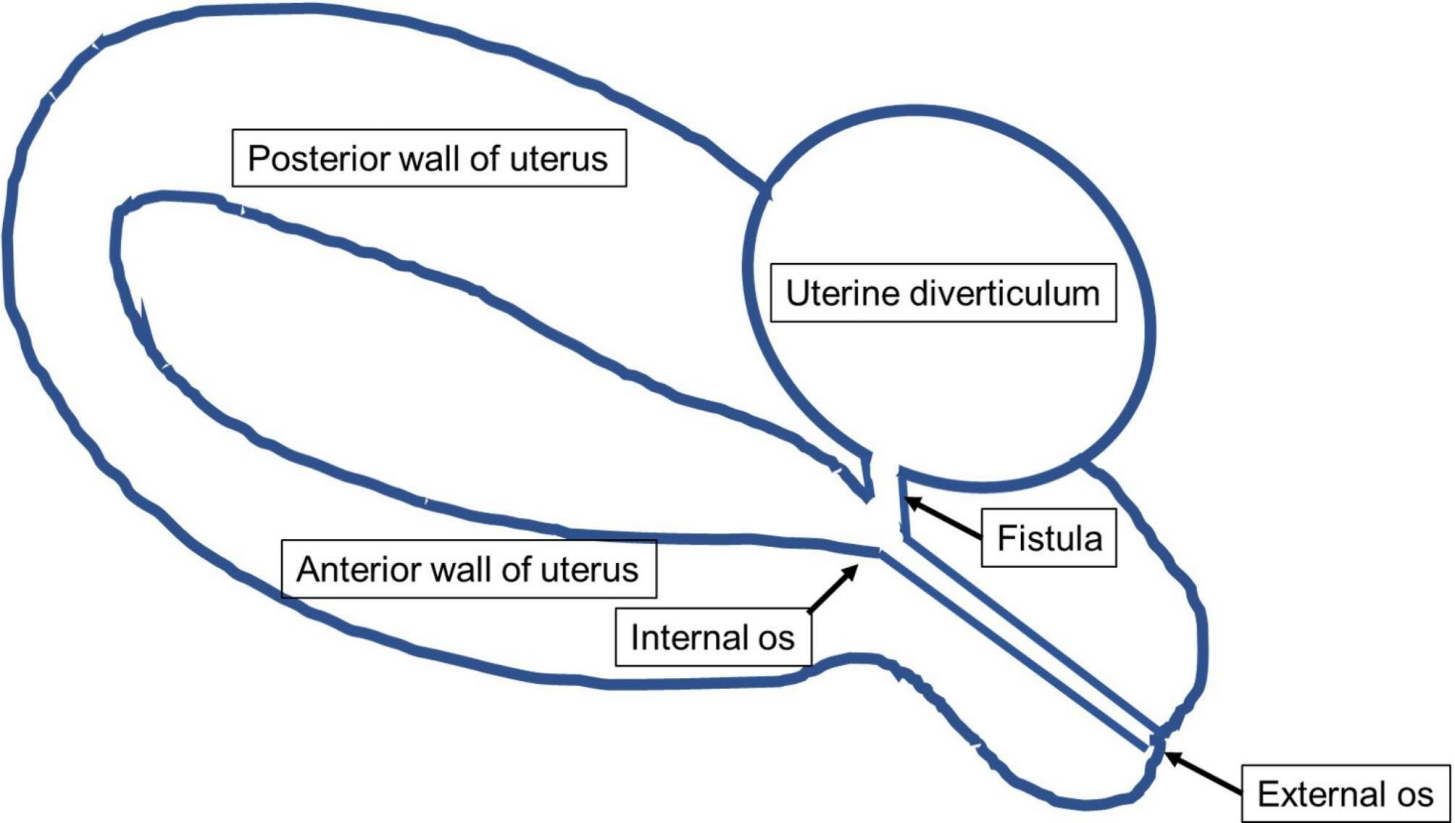
Diagram of the uterus and diverticulum. The diverticulum is on the right side of the posterior wall of the uterus, and a fistula opens near the internal os

**Fig. 3:**
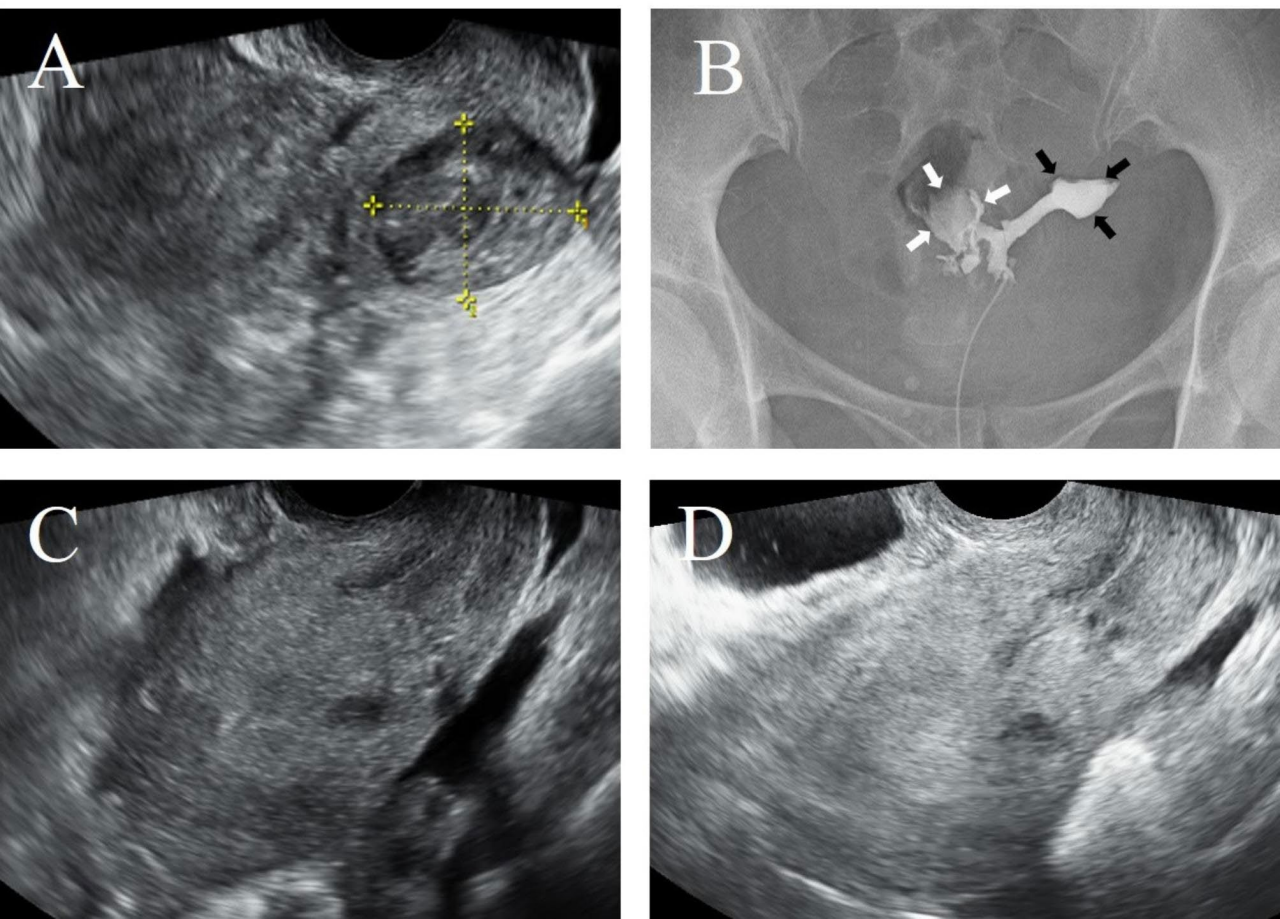
Hysterosalpingography and TV-US findings. **A**: TV-US shows a 3-cm haematoma after myomectomy. **B**: Hysterosalpingography findings; a 3-cm diverticulum containing contrast medium is observed on the right side of the uterus (white arrow), and the uterine body (black arrow) is normal. **C**: TV-US shows the uterus 3 months after haematoma removal. **D**: TV-US shows the uterus 6 months after haematoma removal

During the first visit to our institution, transvaginal ultrasound and MRI showed a 3-cm mass lesion on the posterior wall of the uterus [Figs. 1C and D, 2 and 3A]. Flexible hysteroscopy revealed a hole in the right posterior wall near the internal cervical ostium, and the surrounding regions were white and demarcated. Hysterosalpingography showed a fistula opening slightly above the internal cervical ostium on the posterior wall of the uterus and an approximately 3-cm contrast-enhanced pocket in front of it [Fig. 3B]. Surgery was deemed necessary given the diagnosis of uterine diverticulum following enucleation of a uterine fibroid.

**Fig. 4:**
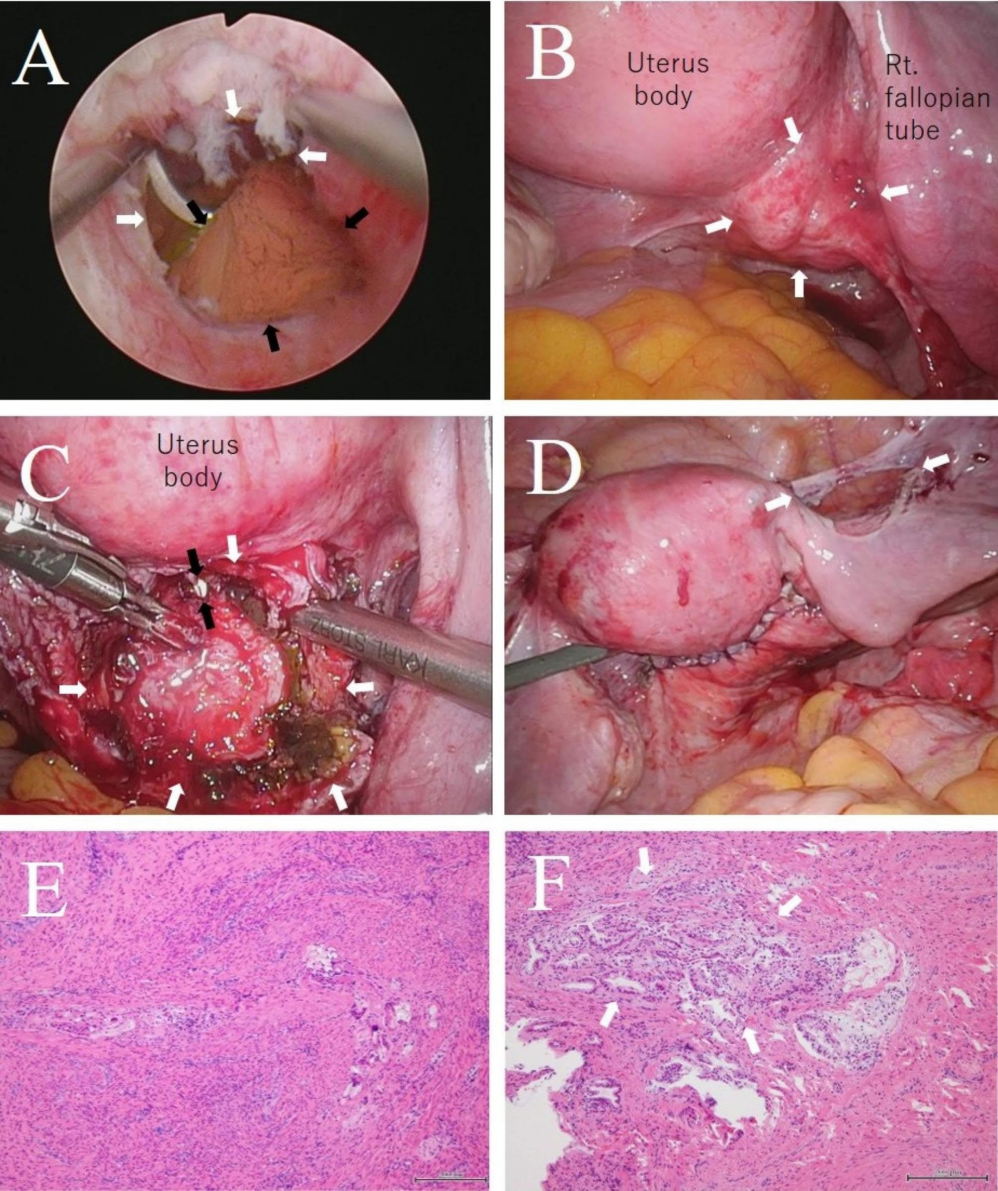
Intraoperative and pathological findings. **A**: Hysteroscopic surgery findings; the entrance of the fistula (white arrow), and accumulated brown blood (black arrow). **B**: Laparoscopic surgery findings; a mass is present on the right side of the lower part of the uterine body (white arrow). **C**: The diverticulum (white arrow) is opened and excised laparoscopically. There is a fistula that continues into the uterine cavity (black arrow). **D**: At the end of the surgery, the stump of the round ligament (white arrow). **E**: Pathological findings, HE staining; multinucleated giant cells appear, and congestion is seen in the myometrium (magnification ×40). **F**: Pathological findings, HE staining; the endometrium invades the myometrium (white arrow) (magnification ×100)

We performed hysteroscopic and laparoscopic diverticulum excision. We deemed it necessary to perform the operation at the earliest time possible, regardless of the menstrual cycle. The procedure was started by employing a hysteroscope. A 5-mm-diameter fistula was observed on the right side of the posterior wall near the internal cervical ostium. A brown blood clot and fluid had accumulated in the pocket [Fig. 4A]. The portion entering the fistula on the internal cervical os side of the demarcated portion was resected, and a manipulator was then inserted into the uterus. The laparoscopic procedure was performed using the diamond port configuration at a pneumoperitoneum pressure of 10 mmHg. The laparoscope showed a 3-cm mass on the posterior wall of the uterus [Fig. 4B]. When the mass was incised and opened, an accumulation of old blood from bleeding inside the mass was observed, and a capsule-shaped cyst had formed. The cyst wall and the normal muscular layer were detached, and the cyst capsule was removed, thereby dissecting the cyst wall from the surrounding uterine tissue. We used ultrasonic energy for dissection, cutting and coagulation (Harmonic Ethicon). A 5-mm-diameter fistula was observed in the posterior portion of the cyst. The tissue around the fistula was trimmed and excised [Fig. 4C]. Using the manipulator as a marker, the fistula was closed with synthetic absorbable thread (Vicryl Ethicon) such that the surrounding muscular layer was brought near the Z suture to cover the fistula closure, and the muscular layer was sutured in three layers [Fig. 4D]. Indigo dye was injected into the uterine cavity, and no flow out of the sutured portion was detected. Finally, fistula closure was confirmed with the hysteroscope. The round ligament was dissected to release the tension, a Nelaton catheter was inserted to prevent cervical stenosis, and the procedure was completed. Histopathologically, the specimen of the incised capsule showed musculo-vascular tissue and suture granuloma formation with haemosiderin phagocytes [Fig. 4E]. Furthermore, endometrial tissues corresponding to the proliferative phase were observed [Fig. 4F]. The postoperative course was good. The patient was discharged on postprocedural Day 6.

Normal menstruation resumed on the 10th day after the procedure, and no abnormal bleeding was observed after the menstrual period. Transvaginal ultrasound performed in the third postprocedure week revealed no indications of mass lesion recurrence. Flexible hysteroscopy showed that the fistula opening near the internal cervical ostium was closed and that the mucosa was smooth. The MRI performed approximately three months after the procedure showed that the layered structure of the uterine muscular layer was maintained [Fig. 1E F], and transvaginal ultrasound showed amelioration of the strong anteflexion of the uterus. No evidence of recurrence was seen on either transvaginal ultrasound, after 3 months [Fig. 3C] and 6 months [Fig. 3D], or flexible hysteroscopy.

## Discussion and conclusion

Most cases with acquired uterine diverticulum have caesarean scar syndrome developing after a caesarean section [1]. Acquired uterine diverticulum after myomectomy has been reported in a few cases, which were all discovered during pregnancy [5]. This is the first report, to our knowledge, to describe diverticulum development after myomectomy in a nonpregnant patient. The prevalence of caesarean scar syndrome after caesarean section ranges from 56 to 80% [6]. A diverticulum reportedly develops in approximately 18% of cases after enucleation of a uterine fibroid [5], and the diverticulum frequency after caesarean section is considered to be high. According to Anna et al., 6 cases (18%) were recognized by hysterosalpingography among 32 cases who had undergone fibroid enucleation [7]. All 6 cases had mucous fibroids, but there were no symptoms or subsequent treatments. This is the first case report describing a post-subserous fibroid nuclear excision patient, with details of the uterine diverticulum. The following are considered to be the causes of uterine diverticulum after caesarean section: (1) incision at a relatively low position near the cervix region; (2) single-layer suture, locking suture, or incomplete closure; and (3) factors that induce the formation of adhesions, such as nonclosure of the peritoneum and improper haemostasis [2][6][8].

In patients who have undergone caesarean section, one of the causative factors might be that the incision site is in the lower uterine segment. The lower uterine segment is potentially weak at the Müllerian duct junction [9]. New blood vessels appear in the surgical scar, and pathologically, bleeding from these new blood vessels and inadequate drainage due to a smaller than normal muscular layer appear to lead to the accumulation of fluid in the scar region. In addition, there might be an endometrial gland in the muscular layer around the scar region, which results in a pathological condition such as adenomyosis.

There are developmental differences between caesarean scar syndrome and postmyomectomy uterine diverticulum. In caesarean scar syndrome, a hysterotomy occurs during pregnancy at the inferior segment of the anterior wall of the uterus with a wound extending into the endometrium. On the other hand, diverticulum after postmyomectomy uterine diverticulum occurs in the nonpregnant state. The location of occurrence and whether the incision reaches the intima depends on the position of the fibroid.

In our present patient, the pedunculated subserosal fibroid was located near the cervix, such that the incision near the cervix had to be sutured. Menstrual blood trapped in the scar is also considered to result in haemorrhage. However, Tanimura et al. reported that such haemorrhage might be caused by the scar itself since they detected an endometrial gland and stromal tissue deep in the scar [2][10]. We also detected endometrial tissue in the incision site, which suggested that the patient’s abnormal bleeding could have been due to bleeding from the capsule tissue itself. Regarding the histopathological findings of the resected diverticulum, not only myometrial tissue but also endometrial glands were found within the myometrium. As previously reported, the cause of irregular bleeding is not only the accumulation of menstrual blood in the diverticulum and its outflow but also bleeding from the endometrial gland that has entered the muscle layer forming the diverticulum. We observed a chocolate-like substance resembling the contents of endometriosis within the cyst both hysteroscopically and laparoscopically. In this case, a small amount of endometrial tissue was pathologically observed in the resected diverticulum, and it is possible that functional bleeding from this site was the cause of the irregular bleeding. There are some reports of endometrial glands and stroma at the site of diverticulum resection in caesarean section scar site syndrome [2, 3, 10, 11]. In the pathological study of cases of total hysterectomy after caesarean section, adenomyosis limited to the scar was observed in 28% of cases, believed to be due to caesarean Sect. [12].

In our case, the pathological examination revealed no endometrial tissue in other resection sites, as in the case of originally existing adenomyotic uteri. The diagnosis of adenomyosis is made histologically, but there are also characteristic findings on ultrasound and MRI [13]. In our case, preoperative MRI and ultrasound were negative for the presence of adenomyosis. Therefore, it was thought that there was a high possibility that the uterine endometrium had infiltrated due to the surgical operation rather than the original adenomyosis.

It is also possible that the endometrium was pulled towards the serosa and sewn in when the pedunculated fibroid was pulled. Histopathologically, the resected specimen of a congenital uterine diverticulum showed a three-layered structure, whereas the histopathological specimen from our present acquired case showed no layered structure. There were also multinucleated giant cells and findings suggestive of marked congestion. These were thought to have been caused by surgical manipulation. In caesarean scar syndrome, which is thought to have a similar pathophysiology, there are no data showing a correlation between the timing of menstrual onset and the incidence of diverticula. However, we anticipate that the correlation between the resumption of menstruation after surgery and the appearance of diverticula will be further elucidated with the accumulation of more cases in the future.

Laparoscopic repair of uterine diverticulum was first reported in 2008 [2] and has since been combined with hysteroscopy. With the latter approach, the scar tissue is excised under a hysteroscope, and the wound is trimmed and sutured employing a laparoscope [3][4]. By adding hysteroscopic surgery to laparoscopic surgery, it is possible to resect the superior and inferior margins of the cervical fistula opening, which are difficult to observe and resect by laparoscopy alone. In addition, hysteroscopy can confirm surgical repair after laparoscopic resection and suturing. This is considered to reduce the risk of complications [14]. In our present case, the tissue around the fistula entrance was resected under hysteroscopy. Furthermore, after laparoscopic diverticulum resection and suturing, the wound was confirmed under hysteroscopy. More recently, there have been reports on the levonorgestrel intrauterine system [6] and low-dose oestrogen plus progestin [14] as hormone therapy. Hormone therapy was not adopted in our present case due to the patient’s desire to have children.

In caesarean scar syndrome, especially in cases with a retroverted uterus, tension is exerted on the wound, and this has been speculated to lead to uterine diverticulum development. The failure rate of repair procedures is high in retroverted uterus cases [6], at approximately 86% [2]. Therefore, in a case with a retroverted uterus, in which lesion excision/suture is not possible, the round ligament can be sewn to reduce the tension exerted on the wound. In our present patient, the wound was on the posterior wall, which was opposite the wound observed in caesarean scar syndrome cases. Since the uterus was markedly anteflexed, it would have exerted strong tension on the wound in the posterior wall, which might have delayed wound healing as well as caused recurrence. Therefore, the round ligament that was detached when searching for the ureter was not sutured. The uterine anteflexion angle was found to be loose upon comparing the uterine sagittal planes in transvaginal ultrasound images obtained on the 7th and 30th days and at the 3rd and 6th months after the procedure. The release of tension on the wound is suggested to be an important procedure, in addition to sufficient trimming of the demarcated portion and careful suturing.

In parallel with the current increase in the rate of caesarean section worldwide, it is expected that late marriage and childbearing will increase the need for uterine preservation and uterine myomectomy. In the presence of the aforementioned conditions, the course may be similar to that of caesarean scar syndrome after myomectomy. The treatment methods will be similar to those applied to caesarean scar syndrome. Reducing the tension exerted on the wound is potentially useful, in addition to removing the demarcated tissue and resuturing by employing a combination of laparoscopy and hysteroscopy. It was also suggested that uterine flexion repair be carried out with a round ligament and preventive wound reduction. According to a case report of congenital uterine diverticulum, uterine diverticulum enlargement was observed during pregnancy, and this enlargement was presumed to be due to increased intrauterine pressure [14]. In this case, it was also suggested that intrauterine pressure release by the Neraton catheter may have contributed to the clinical presentation and thereby the choice of optimal treatment.

## Data Availability

The data presented in this case report are available from the corresponding author on reasonable request.
